# Further insight into genetic variation and haplotype diversity of *Cherry virus A* from China

**DOI:** 10.1371/journal.pone.0186273

**Published:** 2017-10-11

**Authors:** Rui Gao, Yunxiao Xu, Thierry Candresse, Zhen He, Shifang Li, Yuxin Ma, Meiguang Lu

**Affiliations:** 1 State Key Laboratory for Biology of Plant Diseases and Insect Pests, Institute of Plant Protection, Chinese Academy of Agricultural Sciences, Beijing, China; 2 UMR 1332 BFP, INRA, Univ. Bordeaux, Villenave d’Ornon Cedex, France; 3 School of Horticulture and Plant Protection, Yangzhou University, Yangzhou, Jiangsu, China; Oklahoma State University, UNITED STATES

## Abstract

*Cherry virus A* (CVA) infection appears to be prevalent in cherry plantations worldwide. In this study, the diversity of CVA isolates from 31 cherry samples collected from different orchards around Bohai Bay in northeastern China was analyzed. The complete genome of one of these isolates, ChYT52, was found to be 7,434 nt in length excluding the poly (A) tail. It shares between 79.9–98.7% identity with CVA genome sequences in GenBank, while its RdRp core is more divergent (79.1–90.7% nt identity), likely as a consequence of a recombination event. Phylogenetic analysis of ChYT52 genome with CVA genomes in Genbank resulted in at least 7 major clusters plus additional 5 isolates alone at the end of long branches suggesting the existence of further phylogroups diversity in CVA. The genetic diversity of Chinese CVA isolates from 31 samples and GenBank sequences were analyzed in three genomic regions that correspond to the coat protein, the RNA-dependent RNA polymerase core region, and the movement protein genes. With few exceptions likely representing further recombination impact, the trees various trees are largely congruent, indicating that each region provides valuable phylogenetic information. In all cases, the majority of the Chinese CVA isolates clustering in phylogroup I, together with the X82547 reference sequence from Germany. Statistically significant negative values were obtained for Tajima’s *D* in the three genes for phylogroup I, suggesting that it may be undergoing a period of expansion. There was considerable haplotype diversity in the individual samples and more than half samples contained genetically diverse haplotypes belonging to different phylogroups. In addition, a number of statistically significant recombination events were detected in CVA genomes or in the partial genomic sequences indicating an important contribution of recombination to CVA evolution. This work provides a foundation for elucidation of the epidemiological characteristics and evolutionary history of CVA populations.

## Introduction

*Cherry virus A* (CVA), a member of the genus *Capillovirus* in the family *Betaflexiviridae*, was first described in Germany from a sample of sweet cherry (*Prunus avium*). The CVA genome is a single-stranded, positive-sense RNA molecule of 7,383 nucleotides (nt) in length excluding the 3'-terminal poly (A) tail[1]. The genomic organization is similar to that of *Apple stem grooving virus* (ASGV), the type species of *Capillovirus*, with two overlapping open reading frames (ORFs). ORF1 encodes a large 266 kDa polyprotein consisting of the RNA-dependent RNA polymerase (RdRp) fused in-frame to the coat protein (CP). ORF2 encodes a 52 kDa putative movement protein (MP) [1–2].

CVA is frequent in sweet [2] and sour cherry [3] and is also found, less frequently, in other *Prunus* hosts such as apricot, peach [4], and plum [5–6]. The virus is widely distributed and has been reported from many countries, including Germany [1], India [7], Italy [6], France [8], the United Kingdom [9], Canada [4], Poland [10], Serbia [11], the Czech Republic [12], Japan [13], and China [14–15].

A high frequency of CVA infection in tested cherry trees has been reported in different countries, with infection rates of 54% in India [16], nearly 40% in Canada, Germany, and Poland [4, 10], up to 92% in Japan [13]. In China, CVA was first reported in *Prunus mume* and *Prunus avium* [14–15]. Two subsequent studies reported a detection rate of ~60% in tested sweet cherry leaf samples in some regions of China [17–18]. CVA can be readily transmitted by grafting or other vegetative propagation methods. Despite the high incidence of infection, no potential vector has been identified to date for CVA, and its epidemiology is still poorly understood [2]. At present, there is no clear evidence for any association between CVA infection and specific disease symptoms in any of its hosts [19], but severe disease symptoms are sometimes observed in mixed infections involving CVA and other viruses, without information on a possible contribution of CVA to these symptoms [4, 20].

As is often the case in fruit trees, CVA has frequently been identified in mixed infections with one or more other fruit tree viruses such as *Little cherry virus* 1 or 2 (LChV-1, LChV2), *Prune dwarf virus* (PDV), *Plum bark necrosis and stem pitting-associated virus* (PBNSPaV), *Apple chlorotic leaf spot virus* (ACLSV), *Apricot pseudo chlorotic leaf spot virus* (APCLSV), *Cherry greenring mottle virus* (CGRMV), *Cherry necrotic rusty mottle virus* (CNRMV), and *Prunus necrotic ringspot virus* (PNRSV), so that the symptoms observed, if any, cannot easily be associated with CVA infection alone [1, 3, 6–8, 11, 13, 15, 18]. A previous study of Chinese CVA isolates had shown significant sequence diversity, with nucleotide identity levels ranging between 81 and 98.2% for four isolates [19]. At present, there have been only limited efforts to study the molecular variability of CVA isolates, with most of the previous studies focusing on short, partial sequences of the RdRp and MP genes [7–8, 20]. Recently 75 full-length CVA sequences were assembled from next-generation sequencing data by Kesanakurti et al. (2017) [21], providing a large amount of novel sequence information. However, the genetic diversity of CVA Chinese isolates and the evolutionary relationships between global isolates have not yet been extensively studied.

Sweet cherry is a major fruit crop of increasing economic importance in China. Given the very suitable climatic conditions, 70% of the sweet cherry growing area in China is located in regions surrounding Bohai Bay, which include Shangdong, Liaoning, and Hebei provinces, and the cities of Beijing and Tianjing. Of these, Yantai in Shangdong Province and Daliang in Liaoning Provice are the most important sweet cherry production districts.

In the present study, we performed a broad scale analysis of CVA diversity in the regions surrounding Bohai Bay. We included all CVA sequences available in GenBank to provide a further understanding of the patterns of molecular evolution present within the global CVA population.

## Materials and methods

### Plant material and virus isolates

Surveys were conducted between the months of April and October in 2014–2015 in nine sweet cherry orchards in four different districts of Shandong and Liaoning provinces and in Beijing city across the Bohai Bay region in northeastern China. We collected samples from 58 different sweet cherry trees (seven different varieties). Forty two leaf samples were from trees showing symptoms of leaf shriveling, deformation, rolling, yellow and green mottling, or other virus-like symptoms, while 16 samples were from asymptomatic trees. All samples were tested for the presence of CVA and for the presence of 11 additional viruses via RT-PCR using specific primers (see below). The CVA isolates from 31 trees found to be infected by CVA were further characterized as described below. Details of the geographical origins, collection dates, symptoms, and status of mixed infections with other viruses for the 31 CVA isolates analyzed in the present study are given in Table 1. Sequences of the CVA isolates retrieved from the NCBI GenBank database (www.ncbi.nlm.nih.gov) and used for sequence comparisons and evolutionary analyses are described in S1 Table.

**Table 1:** Detection of mixed infections with other viruses in selected CVA-positive *Prunus avium* RNA samples from regions surounding Bohai Bay in northeastern China.

Sample	Variety	Origin	Other viruses identified in the sample	Leaf symptoms^a^
**ChDL3**	Summit	Dalian, Liaoning		NA
**ChDL4**	Hongyan	Dalian, Liaoning	PNRSV	N
**ChDL5**	Hongyan	Dalian, Liaoning		C+B+G
**ChDL6**	Hongyan	Dalian, Liaoning	PDV	E+B+G
**ChDL7**	Hongyan	Dalian, Liaoning	PDV	A
**ChDL9**	Hongdeng	Dalian, Liaoning	PNRSV	N
**ChTA10**	Hongdeng	Taian, Shandong		N
**ChTA11**	Hongdeng	Taian, Shandong		C+B+G
**ChTA12**	Tieton	Taian, Shandong		C+B+G
**ChBJ14**	Hongdeng	Beijing	PNRSV	D+G
**ChBJ17**	Hongdeng	Beijing	PNRSV	C+B
**ChBJ18**	Unknow	Beijing	PDV	F
**ChBJ22**	Unknow	Beijing	LChV-1	A
**ChBJ23**	Unknow	Beijing	LChV-1	A+F
**ChYT30**	Napoleon	Yantai, Shandong	CGRMV, ACLSV, LChV-1	I
**ChYT31**	Hongdeng	Yantai, Shandong	LChV-1	B+C
**ChYT34**	Zaodaguo	Yantai, Shandong	CGRMV, LChV-1	I
**ChYT35**	Ukraine	Yantai, Shandong	CGRMV, LChV-1	I
**ChYT36**	Lapins	Yantai, Shandong	CGRMV, PNRSV, LChV-1	A+B
**ChYT37**	Lapins	Yantai, Shandong	PNRSV, LChV-1	A+B+G
**ChYT38**	Lapins	Yantai, Shandong	PNRSV, LChV-1	A+B+G
**ChYT39**	Lapins	Yantai, Shandong	CGRMV, PNRSV, ACLSV, LChV-1	A+B+G
**ChYT43**	Tieton	Yantai, Shandong	CGRMV, PNRSV, LChV-1	A+B
**ChYT50**	Tieton	Yantai, Shandong	CGRMV, PNRSV	N
**ChYT51**	Sunburst	Yantai, Shandong	CGRMV, PNRSV	A
**ChYT52**	Sunburst	Yantai, Shandong	CGRMV, PNRSV, ACLSV	A+G
**ChYT54**	Summit	Yantai, Shandong	CGRMV, PNRSV, ACLSV	N
**ChYT55**	Tieton	Yantai, Shandong	CGRMV, PNRSV, ACLSV	A+H
**ChYT56**	Sunburst	Yantai, Shandong	CGRMV, PNRSV	N
**ChYT58**	Ukraine	Yantai, Shandong	CGRMV, PNRSV, ACLSV, LChV-1	A+G
**ChYT59**	Ukraine	Yantai, Shandong	CGRMV, PNRSV, ACLSV, LChV-1	A+G

^a^: Symptoms are expressed using the following code: A, yellow and green mottling; B, deformations; C, mosaicism; D, leaf rolling; E, slender leaves; F, cluster; G, crinkling; H, yellow spotting; I, reddening; N, asymptomatic infection; NA, asymptomatic stem but no information on leaf symptoms.

### RT-PCR and cloning, and sequencing

Total RNA was extracted from each cherry sample using the RNAprep Pure Plant Kit (Tiangen Biotech Co., Ltd, Beijing) and subjected to RT-PCR using virus-specific primers. For each isolate, DNA fragments of approximately 1184 bp (positions 5999–7182 on the CVA reference genome), 1195 bp (positions 3698–4892) and 707 bp (positions 5401–6107) corresponding, respectively, to the target domains (plus flanking sequences) of the full-length CP, the core RdRp, and the MP were amplified using the three gene specific primer pairs (S2 Table). These primers were designed based on conserved sequences in the reference CVA genome sequence in GenBank.

First-strand cDNA was synthesized by reverse transcription (RT) at 42°C for 1 h using 1 μl of total RNA and 1 μl of Oligo (dT) primer in a 10 μL reaction with Maloney murine leukemia virus (M-MLV) reverse transcriptase (Promega, Madison, WI, USA), according to the manufacturer’s protocol. Following RT, gene-specific PCR was performed in 15 μl reactions containing 1.2 μl of the cDNA, 7.5 μl of 2X Taq Mix, 5.3 μl distilled water, and 0.5 μl (20 pmol) each of the forward and reverse primers. The thermocycling conditions were as follows: 1 cycle of 5 min at 94°C followed by 35 cycles of 30 s at 94°C, 30 s at 52°C for the CP gene fragment (53°C for the MP gene fragment and 55°C for the RdRp gene fragment), and 90 s at 72°C, with a final extension step of 10 min at 72°C.

To survey the status of mixed infections of the CVA isolates with other viruses, RT-PCR was also used to assay for another 11 viruses (PNRSV, PDV, ACLSV, ApMV, CRLV, CMV, CGRMV, PBNSPaV, CNRMV, LChV-1, and LChV-2) that have been reported to infect cherry in China, using virus-specific primers and the PCR conditions described previously [18].

RT-PCR products were purified using a PCR purification kit (AXygen), and the resulting fragments were ligated into the pGEM-T vector (Takara) and used to transform *E*. *coli* DH5α cells. Plasmid DNA clones that contained the target inserts were identified by colony PCR, and were then sequenced using an automated DNA sequencer (ABI Prism^™^ 3730XL DNA Analyzer). At least three clones of each amplified fragment were sequenced. Sequence reads were assembled using DNAMAN 6.0 (Lynnon Biosoft, Quebec, Canada).

### Determination of the complete genome sequence of the ChYT52 CVA isolate

Four pairs of PCR primers that direct amplification of fragments that span the entire CVA genome (S3 Table) were designed based on the reference CVA sequence (GenBank X82547) and conservative rigions of available genomes in GenBank. The 3′-terminal region was amplified using an oligo (dT) primer and a sequence-specific primer, and the 5′-terminal region was amplified using the 5′-Full RACE Kit with TAP (TaKaRa, Beijing, China), according to the manufacturer’s instructions. RT-PCR was performed as described above, except that we used the 2X Long Taq MasterMix, annealing was for 30 s at 54–56°C, and extension was at 72°C for 150 s. All amplification products were cloned and sequenced as described above. To overcome problems linked to intra-isolate sequence diversity and to avoid mistakes in sequence assembly, adjacent amplicons were designed to overlap for >100 bp, and at least three clones of each PCR product were sequenced. The resulting sequences were then assembled into a single contiguous genomic ChYT52 CVA isolate sequence.

#### Sequence processing

Three to eight clones of each gene fragment from each CVA isolate were sequenced. A total of 125 CP gene sequences, 88 RdRp domain sequences, and 85 MP gene sequences were thus generated from the 31 CVA-infected trees used in this study. These were used together with the corresponding CVA sequences (93 CP, 87 RdRp, and 94 MP genes) available in GenBank to generate the three CP, RdRp, and MP datasets (Table 2, S4–S6 Tables).

**Table 2:** Metrics for sequences used in the analyses in this study.

Gene region	CP			RdRp			MP		
	n ^a^	H ^b^	h ^c^	n	H	h	n	H	h
**This study**	125	65	75	88	64	73	85	59	67
**Sequences available in GenBank**	93	63+2	75	87	69+1	74	94	57+2	73
**Total**	218	128	150	175	133	147	179	116	140

^a^: Number of sequences;

^b^: Number of haplotypes. The number after ‘+’ indicate the number of the same haplotypes with this study;

^c^: Sum of selected haplotypes in this study.

All sequences were aligned with ClustalW as implemented in BioEdit (Hall, 1999) [22]and were edited to remove flanking sequences, leaving only the portions of the alignment coding for the core protein motifs of the CP, MP and RdRp domains available for all isolates, leaving alignments of 615 bp (positions 6466–7080 on the CVA reference genome), 810 bp (positions 3889–4698) and 579 bp (positions 5427–6005) for the CP, core RdRp, and MP datasets, respectively. To ensure that the alignments were in frame, nucleotide sequences were aligned by codon using the ClustalW algorithm implemented in MEGA5 [23]. Conserved regions were determined using the Gblocks program [24]. The aligned sequences were used for the recombination and haplotype analyses. Analysis of the number of haplotypes was performed with DnaSP 5.0 [25]. Following that step, if two or more gene sequences derived from the same tree were identical, they were considered to be a single haplotype and a single sequence was preserved. Final datasets of 150 CP gene sequences, 147 RdRp core sequences, and 140 MP gene sequences, which were subsequently used for phylogenetic and genetics diversity analyses (Table 2, S4–S6 Tables). Some sequences were deposited in the GenBank database under accession numbers NO: KY861857 to KY861925, MF991126 to MF991134 (S7 Table).

### Recombination analysis

The aligned sequences were checked for potential recombination events using the RDP [26], GENECONV [27], BootSCan [28], MaxChi [29], Chimaera [30], SiSCan, and 3Seq programs implemented in the RDP 4.0 software [31]. Potential recombination events detected were considered to be statistically significant if detected by at least four programs with *P* values<10^−6^ [32]. All analyses were performed using the RDP 4.0 default settings for the different programs and a Bonferroni-corrected *P* value cutoff of 0.05 or 0.01.

### Phylogenetic analyses

To investigate the evolutionary history of CVA isolates, phylogenetic analyses were conducted using the various datasets and the neighbour-joining (NJ) methods implemented in MEGA5, with branch stability estimated using 1000 bootstrap replicates. The evolutionary history was inferred by using the NJ method based on Kimura 2-parameter models. Evolutionary analyses for the entire CVA population and for the individual clusters were also conducted in MEGA5 [23].

### Genetic diversity analysis and neutrality tests

To examine the genetic variation of the CVA CP gene, RdRp core, and MP gene, we computed several genetic parameters for each, including haplotype/nucleotide diversity (*H*_d_/*P*_i_) and neutrality (Tajima’s *D* [33] and Fu and Li’s F* [34]) using DnaSP 5.0 [25]. Haplotype diversity refers to the frequency and number of haplotypes in the population and was analyzed using the default settings in DnaSP 5.0. DnaSP implements statistical methods to infer haplotype phase, and prepares adequately the phased data for subsequent analyses. DnaSP reconstructs the haplotype phase by applying various algorithms (PHASE v2.1, fastPHASE v1.1 and HAPAR) differing in the underlying population genetic assumptions [25]. Nucleotide diversity estimates the average pairwise differences among sequences, based on all sites. Tajima’s *D* test is based on the differences between the numbers of segregating sites and the average number of nucleotide differences. Fu and Li’s F* statistical test is based on the differences between the number of singletons and the average number of nucleotide differences between pairs of sequences.

## Results

### RT-PCR detection of CVA infection in Chinese cherry orchards

Cherry orchards in Shandong and Liaoning provinces and Beijing city, around Bohai Bay in northeastern China, were surveyed during the months of April to October in 2014 and 2015. Leaves showing symptoms of shriveling, deformation, rolling, and yellow and green mottling were observed on some of the trees in these orchards. We collected 58 symptomatic or asymptomatic leaf and bark samples from different trees (42 symptomatic trees, 16 asymptomatic ones). RT-PCR analysis [18] showed a high detection rate of CVA infection in tested samples, with 42 out of 58 (72.4%) of the cherry samples testing positive for the presence of CVA. Among these, 31 CVA-positive samples were selected for amplification of three genomic regions corresponding to the CP, RdRp core, and MP domains. The expected amplification DNA fragments (1184 bp, 1195 bp, and 707 bp, respectively) were successfully amplified from all 31 samples. Using RT-PCR methods described previously [18], the status of mixed infections was surveyed in those samples for the following 11 additional viruses: CGRMV, PNRSV, ACLSV, LChV-1, LChV-2, ApMV, CRLV, PDV, CMV, CNRMV, and PBNSPaV. In 26 of the tested 31 samples, in addition to CVA, we detected the presence of between one and four additional viruses (Table 1). The viruses found were PNRSV (16 samples, 51.6% infection), CGRMV (13 samples, 41.9%), LChV-1 (12 samples, 38.7%), ACLSV (6 samples, 19.3%), and PDV (3 samples, 9.6%) (Table 1).

### Haplotype diversity in individual CVA samples

A total of 125 CP gene sequences, 88 RdRp core, and 85 MP gene sequences were generated from the 31 selected CVA-infected cherry RNA samples. In addition to these sequences, we included the CVA sequences (93 CP, 87 RdRp, and 94 MP sequences) retrieved from GenBank (Table 2, S4–S6 Tables) to obtain the datasets used for analysis of haplotype diversity with DnaSP 5.0. We found considerable haplotype diversity among the sequences derived from each sample analyzed in the present study. For the CP domain, haplotype distribution analysis showed that more than one haplotype was found in all but six samples (ChTA10, ChYT34, ChYT43, ChYT52, ChYT55, and ChYT56). We detected a total of 65 distinct haplotypes among the 125 cloned CP PCR fragments. For the RdRp core region, more than one haplotype was also found in each analyzed sample, with the exception of six (ChDL4, ChDL6, ChBJ14, ChYT39, ChYT56, and ChYT58), and 64 distinct haplotypes were found among the 88 RdRp clones sequenced. For the MP gene, we again found more than one haplotype per sample, with the exception of five (ChBJ17, ChYT38, ChYT43, ChYT56 and ChYT59), and there were 59 distinct haplotypes among the 85 MP clones sequenced. The ChYT56 tree is the only one for which only a single haplotype was detected in all three genes. In general, different haplotype frequencies were observed for the three genes. Hap_5 (14 sequences from 6 samples) had the highest frequency for CP haplotypes (21.5%, S4 Table). In order to better analyze each sampled tree, identical haplotypes derived from the same tree were merged. In this way, datasets of 75, 73, and 67 haplotypes were generated for the CP, RdRp, and MP regions, respectively, and with datasets of 87, 74, and 73 haplotypes were generated in the same way for the CP, RdRp, and MP regions retrieved from GenBank, respectively were used in the subsequent evolutionary analyses (Table 2, S4–S6 Tables).

### Determination and analysis of the complete genome sequence of isolate ChYT52

The full-length genome sequence of the ChYT52 CVA isolate was determined from overlapping PCR fragments as described in Materials and Methods. It is 7,434 nt in length, excluding the poly (A) tail. The sequence was deposited in GenBank under accession number KX370827. The genome organization of ChYT52 is identical to that in other CVA isolates with the typical two overlapping ORFs. ORF1 (nt 107–7135) encodes a polyprotein that includes the RdRp (nt 3941–4750) and CP (nt 6518–7132) domains, and the overlapping ORF2 (nt 5452–6843) encodes the MP.

Comparison of the ChYT52 sequence with the 85 complete CVA sequences available in GenBank (S1 Table) showed that the ChYT52 isolate has of 79.9–98.7% nucleotide identity with them. It is however more divergent in the RdRp core, with nucleotide identity levels of 79.1–90.7%, this divergence likely marking a recombination event (see below). However, the encoded proteins show higher levels of sequence conservation (Table 3).

**Table 3:** Comparison of the ChYT52 isolate with 85 complete CVA genomic sequences in GenBank in different genomic regions.

Isolate	Genome	RdRp		MP		CP	
	nt%	nt%	aa%	nt%	aa%	nt%	aa%
**ChYT52 to 85 genomes**	79.9–98.7	79.1–90.7	92.2–99.2	89.1–99.1	96.3–98.9	86.0–99.1	96.5–98.5

RdRp: RNA-dependent RNA polymerase core (genome positions 3889–4698 on the CVA reference genome); MP: movement protein domain (reference genome positions 5427–6005); CP: coat protein domain (reference genome positions 6466–7080).

### Recombination analysis

The presence of recombination events in the CVA genomes, CP, RdRp core, and MP data sets was evaluated using the seven recombination detection programs implemented in RDP 4.0. The identification of candidate recombination events was based on the threshold levels described in Materials and Methods. At least four out of the seven methods predicted recombination events for eight CVA genomes sequences (Table 4), while 2, 7 and 6 putative recombination events were detected in the RdRp, MP and CP datasets, respectively (S8 and S9 Tables). Of the eight recombination events that were detected using the full genomic sequences dataset, one involved ChYT52 and likely explains its higher divergence in the RdRp core region. We also observed that parental sequences of these recombinants could come from the same or different host species. For example, parental sequences for recombinant ChYT52, were identified from *P*. *mume* and *P*. *serrulata*. Four recombinants were detected to possess two crossover sites, but only one site is considered authentic recombination event based on threshold levels described (Table 4).

**Table 4:** Putative recombination events detected in the CVA complete genome dataset using RDP 4.0.

Recombination sequences	Parental sequences		Breakpoint nt)		*p-Value* for the seven detection methods in RDP4*						
	Major	Minor	Begin	End	RD	GE	Bo	Ma	Ch	Si	3S
**KX370827_ChYT52_*P*. *avium***	KY286055_OC_*P*. *mume*	KY510851_13C211_N11_*P*. *serrulata*	5642	158	9.147×10^−17^	1.804×10^−3^	8.410×10^−10^	9.951×10^−10^	1.687×10^−7^	2.177×10^−9^	4.569×10^−10^
	KY510861_13C224_N4_*P*. *serrulata*	KY510891_13C290_N4_*P*. *serrulata*	354	645	9.075×10^−11^	1.542×10^−2^	1.183×10^−9^			2.293×10^−4^	5.102×10^−1^
**KY510909_13TF120_N9_*P*. *avium***	KY286055_OC_*P*. *mume*	KY510851_13C211_N11_*P*. *serrulata*	5725	179	9.147×10^−17^	1.804×10^−3^	8.410×10^−10^	9.951×10^−10^	1.687×10^−7^	2.177×10^−9^	4.569×10^−10^
	KY510861_13C224_N4_*P*. *serrulata*	KY510891_13C290_N4_*P*. *serrulata*	180	629	9.075×10^−11^	1.542×10^−2^	1.183×10^−9^			2.293×10^−4^	5.102×10^−1^
**KY510861_13C224_N4_*P*. *serrulata***	LC125634_J_*P*. *armeniaca*	KY510862_13C224_N6_*P*. *serrulata*	7427	89	8.440×10^−7^	6.948×10^−9^	1.056×10^−7^			3.073×10^−2^	
**KY510845_13C202_N10_*P*. *cerasus***	KY510882_13C260_N11_*P*. *avium*	KY510848_13C206_N13_*P*. *serrulata*	5465	7431	9.827×10^−156^	1.000×10^−151^	7.601×10^−73^	5.942×10^−33^	5.896×10^−33^	4.413×10^−38^	4.407×10^−178^
**KY510857_13C217_N3B_*P*. *avium***	KU215410_Lambert_43_*Cherry*	KY510886_13C287_N10_*P*. *serrulata*	7351	7403	2.676×10^−48^	2.438×10^−44^	2.648×10^−48^	1.026×10^−7^	1.020×10^−7^	3.994×10^−14^	
**KY510859_13C222_N8_*P*. *avium***	KY510891_13C290_N4_*P*. *serrulata*	KY510845_13C202_N10_*P*. *cerasus*	4884	5463	1.067×10^−12^	7.463×10^−1^	5.695×10^−14^	5.049×10^−7^	2.568×10^−8^	7.470×10^−8^	1.038×10^−5^
	KY510890_13C290_N2_*P*. *serrulata*	KT285841_ChTA11_*P*. *avium*	6988	7436	3.412×10^−6^	4.650×10^−9^	7.132×10^−8^	6.126×10^−1^	4.859×10^−2^	7.444×10^−3^	
**KY510862_13C224_N6_*P*. *serrulata***	KY510891_13C290_N4_*P*. *serrulata*	KY510845_13C202_N10_*P*. *cerasus*	4888	5467	1.067×10^−12^	7.463×10^−1^	5.695×10^−14^	5.049×10^−7^	2.568×10^−8^	7.470×10^−8^	1.038×10^−5^
	KY510890_13C290_N2_*P*. *serrulata*	KT285841_ChTA11_*P*. *avium*	6993	95	3.412×10^−6^	4.650×10^−9^	4.336×10^−7^			7.444×10^−3^	
**KY510866_13C231_N4_*P*. *serrulata***	KY510911_13TF127_N29_*P*. *avium*	KY510857_13C217_N3B_*P*. *avium*	5993	7345	1.260×10^−56^	2.817×10^−11^	3.688×10^−56^	7.172×10^−16^	1.739×10^−17^	2.751×10^−12^	1.111×10^−31^
**KY510893_13TF101_N34_*P*. *avium***	KY510903_13TF114_N5_*P*. *avium*	KY510909_13TF120_N9_*P*. *avium*	6342	7431	9.031×10^−83^	8.470×10^−80^	2.984×10^−80^	1.592×10^−18^	8.942×10^−19^	1.978×10^−21^	1.367×10^−54^

*: *P-values* determined for each of the seven different programs (RD: RDP, GE: GENECONV, Bo: BootScan, Ma: MaxChi, Ch: Chimaera, Si:SiSCan, 3S: 3Seq) implemented in RDP 4.0 software.

### Phylogenetic analyses

Using the full-length (FL) genomes, CP, RdRp, and MP datasets, we calculated the average pairwise genetic distances (nucleotide diversities). The overall mean nucleotide diversities were 0.171±0.003 for the FL genomes, 0.095±0.007 for the 150 CP sequences, 0.131 ±0.006 for the 147 RdRp sequences, and 0.076±0.007 for the 140 MP sequences.

**Fig 1:**
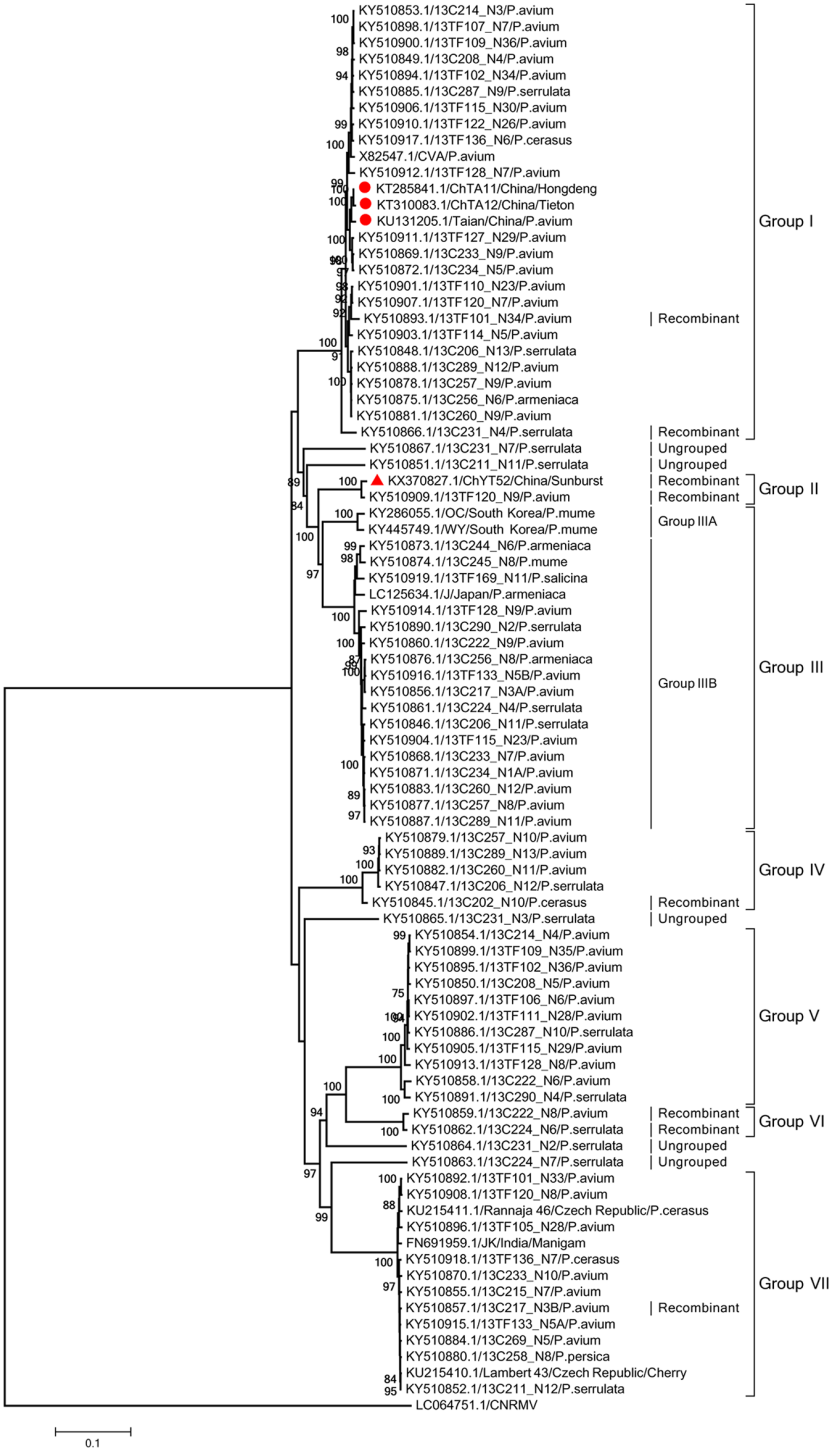
Phylogenetic analysis of CVA isolates based on complete genome sequence. Trees were constructed by the neighbor-joining (NJ) method using MEGA5 software. Bootstrap values (1,000 replicates) are given at the branch nodes. Branches corresponding to partitions reproduced in <75% of bootstrap replicates are collapsed. The isolate for which the complete genome sequence was obtained in the present study (ChYT52) is marked with a red triangle while sequences from Chinese obtained in GenBank are marked with a red round. *Cherry necrotic rusty mottle virus* (CNRMV) was used as out-group.

The phylogenetic analysis of the FL genomic sequences, including ChYT52, clustered sequences into seven major phylogroups (designated I to VII, Fig 1). In addition, 5 non-recombinant isolates remained unclustered at the end of long branches, indicating the likely existence of further phylogroup diversity in CVA. Chinese isolate ChYT52 forms a separate phylogroup (Group II) with the 13TF120_N9 isolate, while the three other previously sequenced Chinese isolates (ChTA11, ChTA12, and Tai’an) are closely related and cluster in Phylogroup I (Fig 1). The majority of non-cherry isolates cluster in Phylogroup III, together with some cherry isolates. However, two non-cherry isolates are found in Phylogroup I (KY510875) and Phylogroup VII (KY510880) (Fig 1).

**Fig 2:**
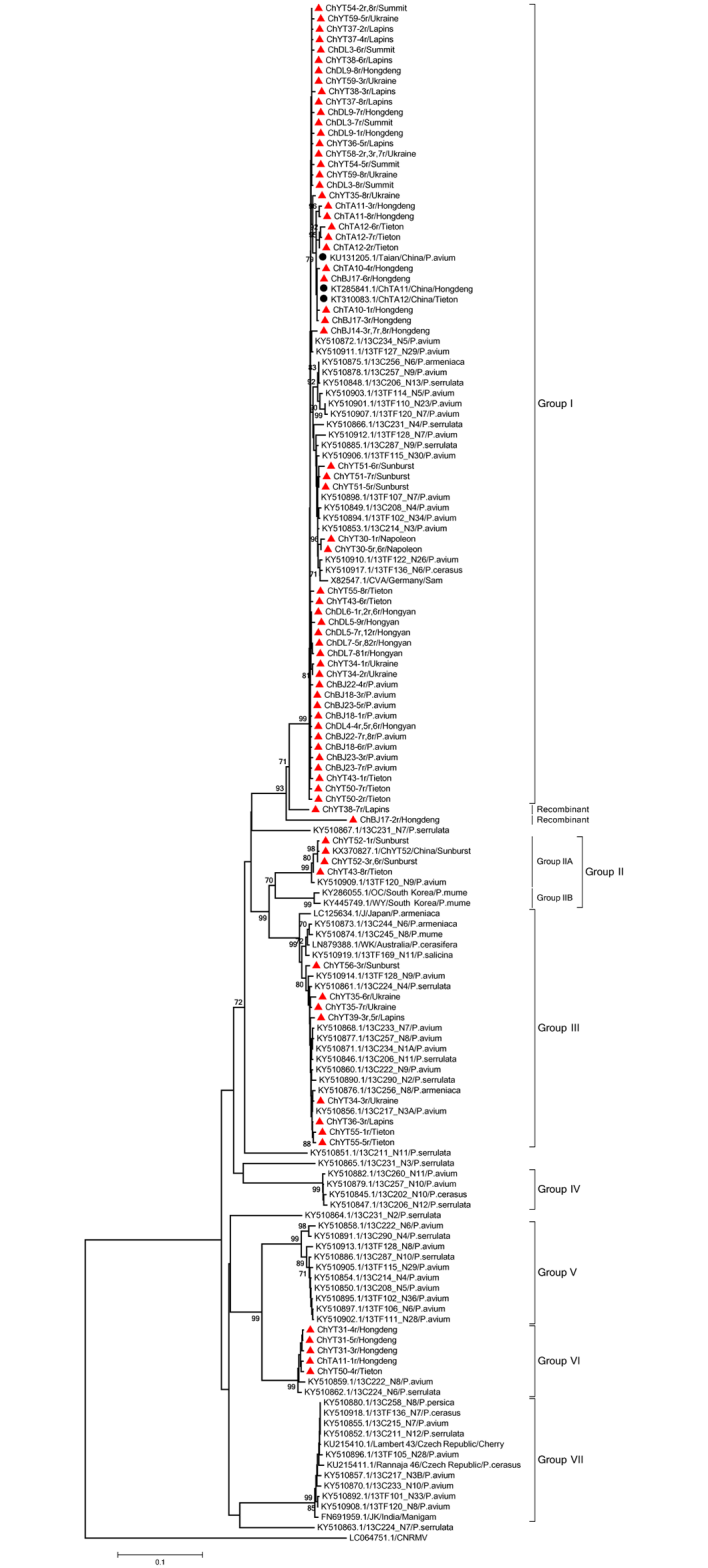
Phylogenetic analysis of CVA isolates based on nucleotide sequence datasets of the RdRp core region. Trees were constructed by the neighbor-joining (NJ) method using MEGA5 software. Bootstrap values (1,000 replicates) are given at the branch nodes. Branches corresponding to partitions reproduced in <70% of bootstrap replicates are collapsed. Sequences obtained in this study are marked with a red triangle while those Chinese isolates from GenBank are marked with a black round. *Cherry necrotic rusty mottle virus* (CNRMV) was used as out-group.

**Fig 3:**
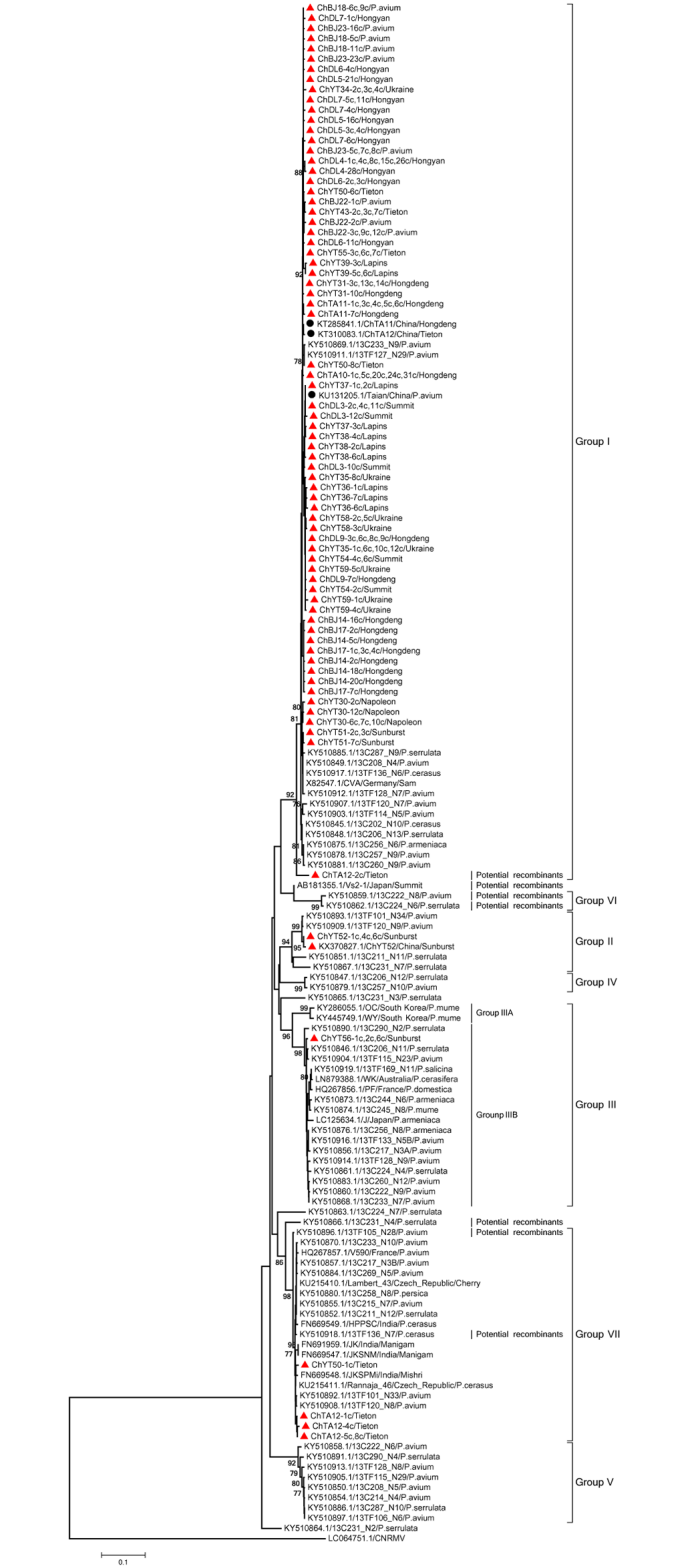
Phylogenetic analysis of CVA isolates based on nucleotide sequence datasets of the CP conserved domain. Trees were constructed by the neighbor-joining (NJ) method using MEGA5 software. Bootstrap values (1,000 replicates) are given at the branch nodes. Branches corresponding to partitions reproduced in <75% of bootstrap replicates are collapsed. Sequences obtained in this study are marked with a red triangle while those Chinese isolates from GenBank are marked with a black round. *Cherry necrotic rusty mottle virus* (CNRMV) was used as out-group.

**Fig 4:**
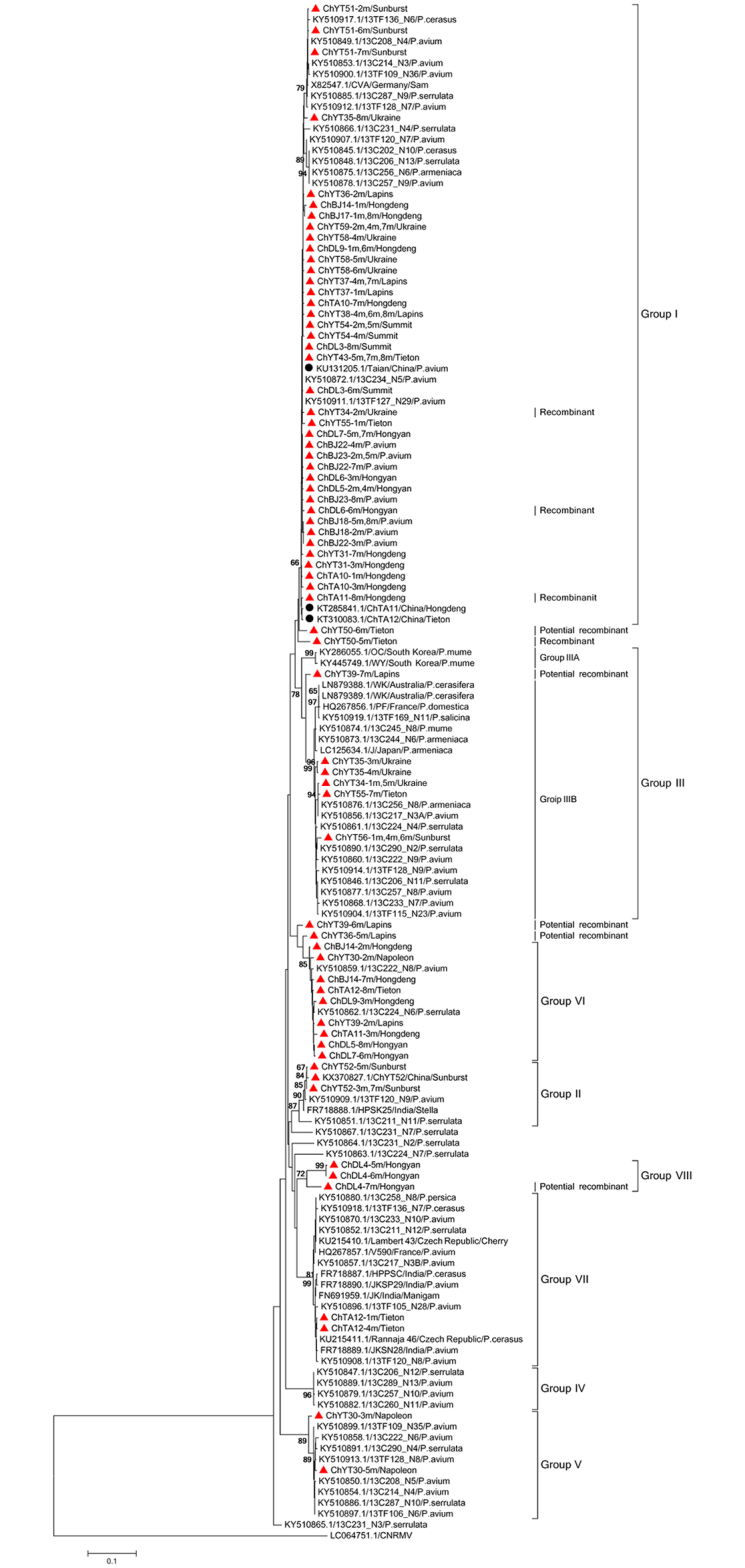
Phylogenetic analysis of CVA isolates based on nucleotide sequence datasets of the MP conserved domain. Trees were constructed by the neighbor-joining (NJ) method using MEGA5 software. Bootstrap values (1,000 replicates) are given at the branch nodes. Branches corresponding to partitions reproduced in <65% of bootstrap replicates are collapsed. Sequences obtained in this study are marked with a red triangle while those Chinese isolates from GenBank are marked with a black round. *Cherry necrotic rusty mottle virus* (CNRMV) was used as out-group.

The phylogenetic trees reconstructed with the other three datasets (Figs 2–4) are very largely congruent with the tree, with few originalities. Among the few differences observed is the homogeneity of Phylogroup III in the RdRp tree when sub-clustering in two distinct, bootstrap supported subgroups (IIIA and IIIB, Fig 1) can be observed in the FL, MP and CP trees. The reverse situation occurs for Phylogroup II, which is homogenous in the latter trees but show sub-structuring in the RdRp tree (Fig 2). Given the importance of recombination events in the evolutionnary history of CVA identified above, it is likely that most of the incongruences between the different phylogenetic trees are the consequence of such recombination events.

The largest diversity of Chinese isolates is observed in the MP tree. While most chinese sequences cluster in the large Phylogroup I, sequences belonging to Phylogroups II, III, V, VI and VII are also observed. In addition, sequences from a unique tree, ChDL4 (ChDL4-5, -6 and -7, Fig 4) do not appear to have any close relative and may represent yet another Phylogroup, for which no FL sequence is available. In contrast the diversity of Chinese isolates observed in the RdRp or CP trees is lower, with not representatives of Phylogroups V and VII in the RdRp tree and no representatives of groups V and VI in the CP tree, respectively.

Mixed infections of single trees by CVA sequence variants belonging to different phylogenetic groups were observed in several cases. For instance, among the CP gene sequences determined here, two isolates (ChYT50, ChTA12) contained sequence haplotypes belonging to two different phylogroups (Figs 2–4). Two haplotypes from the ChYT50 tree belong to Group I while another haplotype belongs to Group VII. Similarly, three haplotypes from ChTA12 belong to Group VII while a GenBank sequence previously determined from this tree belongs to Group I. Haplotypes belonging to different phylogroups were observed from nine trees for the RdRp gene (ChTA11, ChBJ17, ChYT34, ChYT35, ChYT36, ChYT38, ChYT43, ChYT50, ChYT55) and from thirteen trees (ChDL4, ChDL5, ChDL7, ChDL9, ChTA11, ChTA12, ChBJ14, ChYT30, ChYT34, ChYT35, ChYT36, ChYT39, ChYT55) for the MP gene (Figs 2–4).

### Genetic diversity analysis and neutrality tests

DnaSP 5.0 was used to calculate the haplotype diversity (*H*_d_), to estimate the nucleotide diversity (*P*_i_), and to perform Tajima’s *D* and Fu and Li’s F* statistical tests of neutrality on the phylogroups from each gene dataset. *H*_d_ values for the MP, CP, and RdRp gene sequences were found to range from 0.8333–1.000, and the *P*_i_ values were found to be <0.0470 in each group, confirming that a high level of genetic diversity is present in the CVA population. To determine the influence of demographic forces on each gene-specific data set, we calculated Tajima’s *D* values. Negative values with statistical significance (P<0.05 or P<0.01) were obtained for Tajima’s *D* only for Group I for the RdRp, CP and MP datasets, and for Group VII with the RdRp and CP datasets, and these results were further confirmed by Fu and Li’s F* statistical test values (Table 5), suggesting that these two group may be undergoing an expansion phase. On the contrary, given the low number of sequences in Phylogroups II, III, IV, V and VI, no statistically significant negative or positive values were obtained for the statistical tests, suggesting that these subpopulations may be undergoing a neutral or contraction period or, alternatively, that the number of sequences is too limited to give sufficient statistical power to the estimation. Statistical tests for the FL genomes dataset were also performed, which no statistically significant negative values were obtained on the all phylogroups (data not shown).

**Table 5:** Haplotype/nucleotide diversity and neutrality tests in three different genomic regions with respect to phylogroups of CVA isolates.

Region	Group	*n*	*h*	*H*_d_	*P*_i_	Tajima’s *D*	Fu and Li’ F*
**RdRp**	Global	147	133	0.997	0.1134	1.2223	-0.2338
	I	77	66	0.991	0.0122	-2.1953**	-4.5416**
	II	7	7	1.000	0.0470	0.6477	1.0325
	III	23	23	1.000	0.0118	-1.8122*	-2.5073
	IV	4	4	1.000	0.0051	-0.4464	-0.4394
	V	10	10	1.000	0.0132	-0.6573	-0.5074
	VI	7	7	1.000	0.0078	-1.2913	-1.5556
	VII	12	9	0.909	0.0057	-1.8517*	-2.4334*
**CP**	Global	150	128	0.996	0.0845	0.6791	-0.9929
	I	86	70	0.992	0.0112	-2.1733**	-4.6511**
	II	6	6	1.000	0.0385	-0.6384	-0.6973
	III	20	19	0.995	0.0255	-1.1676	-0.4637
	IV	2	2	1.000	0.0081	n.d.	n.d.
	V	8	7	0.964	0.0113	-0.9375	-0.9879
	VI	3	3	1.000	0.0472	n.d.	n.d.
	VII	21	17	0.967	0.0065	-1.9398*	-2.6835*
**MP**	Global	140	116	0.995	0.0696	0.1101	-0.9793
	I	57	42	0.981	0.0099	-1.8564*	-3.8539**
	II	6	6	1.000	0.0176	-1.2461	-1.4305
	III	25	22	0.990	0.0191	-1.5092	-1.3576
	IV	4	3	0.833	0.0017	-0.7099	-0.6043
	V	10	8	0.933	0.0080	-1.4609	-1.9121
	VI	11	11	1.000	0.0088	-1.7351	-1.9735
	VII	16	13	0.950	0.0075	-1.2626	-1.8084
	VIII	3	3	1.000	0.0449	n.d.	n.d.

*n*: number of sequences, *h*: number of haplotypes, *H*_d:_ haplotype (gene) diversity, *P*_i:_ nucleotide diversity (per site), n.d.: not determined (fewer than four sequences),

*: P<0.05,

**: P<0.01,

***: P<0.001

## Discussion

Wang et al.(2013) [17] reported the identification of LChV-1, LChV-2 and CVA on sweet cherry around the Bohai Bay with a CVA detection rate of 60.8% (31/51). But all samples came from the sweet cherry cultivar “Hongdeng”. Lu et al. (2015) [18] also tested a limited number of samples (20) from the Bohai Bay area (but excluding Yantai, the area with the most production), again finding an infection rate of 60%. In the present study, we extended these analyses by including more sweet cherry cultivars and cropping areas around the Bohai Bay. We tested 58 RNA samples isolated from sweet cherry for the presence of 12 stone fruit tree viruses. The virus most frequently detected was CVA (72.4% of tested samples), confirming the above initial evaluations in China, and similar to the high detection levels previously reported in other countries such as India [16], Germany [4] and Japan [13]. The high incidence of CVA likely reflects its efficient transmission through vegetative propagation practices and the absence of efforts to ensure its elimination from cherry multiplication stocks. Whether CVA can also be transmitted from plant to plant by (an) other, as yet unidentified mechanism(s), remains a point of speculation. At the same time, most CVA infections occur as mixed infections with one or several other viruses such as PNRSV, PDV, LChV-1, CGRMV, and ACLSV. This result confirms previous observations [3, 11, 13] and likely explains the variety of symptoms observed on some of the sampled plants.

Molecular evolution studies can help us to understand the important features of RNA viruses, such as population structure, adaptation to new hosts, and the underlying evolutionary mechanisms [35]. Several such studies on viruses in the family *Betaflexiviridae*, such as ASGV [36], *Grapevine virus A* (GVA) [37] and ACLSV [38–39] have recently been published. To date, there have only been limited efforts in the case of CVA, a virus of very broad geographical distribution [14, 20–21]. In the present study, the genetic diversity and evolution of CVA were further analyzed using three different genomic regions representative of the three functional domains of the CP, RdRp, and MP genes using data accessed from international nucleotide sequence databases and from 31 CVA Chinese samples derived from the largest sweet cherry planting regions (70% of the total cherry growing area in China) adjacent to Bohai Bay.

Characterization of the molecular diversity of plant virus populations has also been the aim of an increasing number of studies. This diversity can be analyzed considering different criteria, which provide different levels of information. One of these criteria, used here, is haplotype diversity [40]. In addition, when analyzing haplotypes, the occurrence of recombinant or reassortant viruses can be revealed. For a virus to expand its host range, there must already exist in the viral population (maybe in low proportion) a variant with the ability to infect, maybe with only low efficiency, the new potential host. Recombination may play a significant role in virus survival by reducing the amount of deleterious mutations incorporated in an individual virus genome [41]. Recombination events have also been reported to be evolutionarily important in shaping the genomes of some viruses in CVA, GVA and ACLSV [21, 37, 39].

DnaSP is a software package for the comprehensive analysis of DNA polymorphism data, including haplotype phasing [25]. In recent years, a few studies using DnaSP have reported high haplotype diversity values in plant RNA virus populations [32, 42, 43], but these studies were mostly focused on Tajima’s *D* and Fu and Li’s D* and F* test. In addition, using a threshold of 98 to 100% nt identities among sequence haplotypes, Alabi et al.(2014) determined the number and diversity of haplotypes of *Grapevine virus* A, showing considerable haplotype diversity in individual isolates based on RdRp and CP sequences and the co-existence in infected plants of divergent viral variants [37]. Divergent viral variants have been also reported for ASGV in the family *Betaflexiviridae* [44].

Similarly, in the present study, haplotype number and frequency analysis using DnaSP showed that there was considerable haplotype diversity in the individual genes, and high frequency haplotypes were unfrequent. Indeed, more than one haplotype were observed in each sample except for ChYT56. More than half of the Chinese CVA samples contained genetically diverse haplotypes belonging to different phylogroups, providing conditions suitable for the emergence of recombinant isolates. Indeed, the isolates analyzed here provide evidence of recombination events in the CVA genomes and its three regions (CP, RdRp, and MP genes). Kesanakurti., et al. (2017) [21] have reported the identification of four potential CVA recombinants. The re-alaysis performed here, with a slightly larger dataset allowed to double the number of potential recombinants when analyzing FL genomic sequences. Yet additional recombinants were detected when using the partial RdRp, MP and CP sequences, for which the number of sequences available is significantly larger. In total, six recombination events of Chinese isolates and 13 potential recombination events (7 from Chinese isolates) were identified in the three genomic regions analyzed, further highlighting the contribution of recombination to the evolution of CVA. Another unique observation was that for two of the recombinants identified in the present study, identified parental sequences were from *P*. *mume* and *P*. *serrulata*, suggesting for the first time that recombination may occur between CVA isolates from cherry and non-cherry hosts.

The phylogenetic analyses performed with the different datasets provided largely convergent results, with the identification of 7 Phylogroups and of at least 5 divergent isolates that may represent further phylogroups. The results reported here are largely parallel to those reported in Kesanakurti., et al. (2017). There are however a few minor differences, such as the observation of a 7^th^ phylogroup and the substructuring withing phylogroups II and III. This likely results from the inclusion of the additional sequences reported here. One point worth mentionning is that the 5 divergent isolates standing at the end of long branches are all *P*. *serrulata* isolates, suggesting that further investigations of CVA diversity in *P*. *serrulata* is likely to provide additional insight into CVA diversity. The trees generated using the partial sequences datasets have roughly the same topology as the FL sequences tree, with only in the case of the MP tree possible evidence for the existence of a further phylogroup. This tends to indicate that a large proportion of CVA diversity may by now have been characterized. The majority of the Chinese CVA isolate sequences cluster in the main Phylogroup (Group I) with the reference sequence X82547 from Germany but the results reported here provide evidence for the presence of at least 6 and possibly 7 CVA phylogroups in China, a diversity that has so far no equivalent worldwide. Statistically significant negative values were obtained for Tajima’s *D* and Fu and Li’s F* in Group I for the CP, RdRp, and MP gene sequences, suggesting that this viral group may be undergoing an period of evolutionary expansion.

It should be stressed that even though considerable genomic diversity was identified for CVA in the present study, some further components of CVA diversity may have been missed, because there is no guarantee that the PCR primers used are able to amplify all isolates or that a divergent variant may have been lost by chance when picking the clones for sequencing. This may explain why members of Phylogroups V, VI and VII, detected in the MP tree were not observed in the RdRp (phylogroups V and VII) and in the CP (Phylogroups V and VI) trees. The alternative explanations are either an insufficient sequencing effort of cloned PCR products or the further existence of recombinant isolates. The large and possibly still underestimated diversity of CVA may pose problems in terms of diagnostics, because some isolates may altogether escape detection due to the primers that are currently used in PCR. Besides the fact that it is a relatively young tree, we have no explanation for the observation that ChYT56 is the only tree for which a single haplotype was observed in the three analyzed regions. However, we did performed sRNA NGS analysis of 3 samples in this study (data not shown) and the results obtained did not provide additional information or isolates clusters that had been already detected by PCR. Future studies, involving in particular non-oriented approaches such as NGS (next generation sequencing) will be critical to reveal any additional CVA sequences in the samples tested here and, more broadly, any still undescribed components of CVA diversity.

It is worth noting that in their phylogenetic analyses of the partial RdRp gene of CVA, Marais et al. (2012) [20] identified a cluster of isolates associated with non-cherry hosts. However, in the present study, members of this cluster (Group III) for the three genome regions analyzed were all identified from cherry trees. This cluster can therefore no longer be considered as a group of non-cherry isolates. This observation raises questions about the origin and mode of exchange between cherry and non-cherry hosts for Group III CVA isolates, and also about the mechanisms of adaptation of CVA to these various hosts.

## Supporting information

S1 Table CVA isolate sequences retrieved from GenBank. a: NA, not available; b: CP, coat protein; MP, movement protein; RdRp, RNA-dependent RNA polymerase.
(DOC)

S2 Table Oligonucleotide primers used for amplification of the CP, RdRp, and MP CVA gene regions used in this study. (DOC)

S3 Table Oligonucleotide primers used to amplify the complete genome sequence of the ChYT52 isolate of CVA. (DOC)

S4 Table Number of CP gene-specific haplotypes present in 31 cherry RNA samples analyzed in this study and available isolates in GenBank. (DOC)

S5 Table Number of RdRp gene-specific haplotypes present in 31 cherry RNA samples analyzed in this study and available isolates in GenBank. (DOC)

S6 Table Number of MP gene-specific haplotypes present in 31 cherry RNA samples analyzed in this study and available isolates in GenBank. (DOC)

S7 Table Accession numbers of the different CP, RdRp, and MP clones obtained from GenBank used in this study. (DOC)

S8 Table Recombination events detected in the RdRp and MP gene datasets using RDP 4.0. a: *P-values* determined for eachof the seven different programs (RDP, GENECONV, BootScan, MaxChi, Chimaera, SiSCan, and 3Seq) implemented in RDP 4.0 software.
(DOC)

S9 Table Potential recombination events detected in the CP and MP gene datasets using RDP 4.0. a: *P-values* determined for eachof the seven different programs (RDP, GENECONV, BootScan, MaxChi, Chimaera, SiSCan, and 3Seq) implemented in RDP 4.0 software. *: These isolates may be actual recombinants.
(DOC)
