# Supplementary material for: Further insight into genetic variation and haplotype diversity of Cherry virus A from China
Source: PLoS One. 2017 Oct 11;12(10):e0186273. doi: 10.1371/journal.pone.0186273 (PMC5636130; doi:10.1371/journal.pone.0186273)
Supplement: S1 Table — a: NA, not available; b: CP, coat protein; MP, movement protein; RdRp, RNA-dependent RNA polymerase. (DOC) [file pone.0186273.s001.doc]

**Supporting Information**

**Further Insight to Genetic Variation and Haplotype Diversity of *Cherry virus A* from China**

Rui Gao^1¶^, Yunxiao Xu^1¶^, Thierry Candresse^2^, Zhen He^3^, Shifang Li^1^, Yuxin Ma^1,2^, Meiguang Lu^1^*

^1^ State Key Laboratory for Biology of Plant Diseases and Insect Pests, Institute of Plant Protection, Chinese Academy of Agricultural Sciences, Beijing, China;

^2^ UMR 1332 BFP, INRA, Univ. Bordeaux, CS20032, 33882 Villenave d’Ornon Cedex, France;

^3^ School of Horticulture and Plant Protection, Yangzhou University, Yangzhou, Jiangsu, China.

^¶^These authors contributed equally to this work.

*Corresponding author:

Meiguang Lu ([mglu@ippcaas.cn](mailto:mglu@ippcaas.cn))

**S1 Table.** CVA isolate sequences retrieved from GenBank.

| **Isolate** | **Host** | **Origin^a^** | **Genomic region^b^** | **Accession number** |
| --- | --- | --- | --- | --- |
| **JK** | *P. avium* cv. Manigam | India | Genome | FN691959 |
| **CVA** | *P. avium* cv. Sam | German | Genome | X82547 |
| **ChTA11** | *P. avium* cv. Hongdeng | China | Genome | KT285841 |
| **ChTA12** | *P. avium* cv. Tieton | China | Genome | KT310083 |
| **Taian** | *P. avium* | China | Genome | KU131205 |
| **ChYT52** | *P. avium* cv. Sunburst | China | Genome | KX370827 |
| **Rannaja 46** | *P. cerasus* | Czech Republic | Genome | KU215411 |
| **Lambert 43** | Cherry | Czech Republic | Genome | KU215410 |
| **J** | *P. armeniaca* | Japan | Genome | LC125634 |
| **OC** | *P. mume* | South Korea | Genome | KY286055 |
| **WY** | *P. mume* | South Korea | Genome | KY445749 |
| **13C202_N10** | *P. cerasus* | NA | Genome | KY510845 |
| **13C206_N11** | *P. serrulata* | NA | Genome | KY510846 |
| **13C206_N12** | *P. serrulata* | NA | Genome | KY510847 |
| **13C206_N13** | *P. serrulata* | NA | Genome | KY510848 |
| **13C208_N4** | *P. avium* | NA | Genome | KY510849 |
| **13C208_N5** | *P. avium* | NA | Genome | KY510850 |
| **13C211_N11** | *P. serrulata* | NA | Genome | KY510851 |
| **13C211_N12** | *P. serrulata* | NA | Genome | KY510852 |
| **13C214_N3** | *P. avium* | NA | Genome | KY510853 |
| **13C214_N4** | *P. avium* | NA | Genome | KY510854 |
| **13C215_N7** | *P. avium* | NA | Genome | KY510855 |
| **13C217_N3A** | *P. avium* | NA | Genome | KY510856 |
| **13C217_N3B** | *P. avium* | NA | Genome | KY510857 |
| **13C222_N6** | *P. avium* | NA | Genome | KY510858 |
| **13C222_N8** | *P. avium* | NA | Genome | KY510859 |
| **13C222_N9** | *P. avium* | NA | Genome | KY510860 |
| **13C224_N4** | *P. serrulata* | NA | Genome | KY510861 |
| **13C224_N6** | *P. serrulata* | NA | Genome | KY510862 |
| **13C224_N7** | *P. serrulata* | NA | Genome | KY510863 |
| **13C231_N2** | *P. serrulata* | NA | Genome | KY510864 |
| **13C231_N3** | *P. serrulata* | NA | Genome | KY510865 |
| **13C231_N4** | *P. serrulata* | NA | Genome | KY510866 |
| **13C231_N7** | *P. serrulata* | NA | Genome | KY510867 |
| **13C233_N7** | *P. avium* | NA | Genome | KY510868 |
| **13C233_N9** | *P. avium* | NA | Genome | KY510869 |
| **13C233_N10** | *P. avium* | NA | Genome | KY510870 |
| **13C234_N1A** | *P. avium* | NA | Genome | KY510871 |
| **13C234_N5** | *P. avium* | NA | Genome | KY510872 |
| **13C244_N6** | *P. armeniaca* | NA | Genome | KY510873 |
| **13C245_N8** | *P. mume* | NA | Genome | KY510874 |
| **13C256_N6** | *P. armeniaca* | NA | Genome | KY510875 |
| **13C256_N8** | *P. armeniaca* | NA | Genome | KY510876 |
| **13C257_N8** | *P. avium* | NA | Genome | KY510877 |
| **13C257_N9** | *P. avium* | NA | Genome | KY510878 |
| **13C257_N10** | *P. avium* | NA | Genome | KY510879 |
| **13C258_N8** | *P. persica* | NA | Genome | KY510880 |
| **13C260_N9** | *P. avium* | NA | Genome | KY510881 |
| **13C260_N11** | *P. avium* | NA | Genome | KY510882 |
| **13C260_N12** | *P. avium* | NA | Genome | KY510883 |
| **13C269_N5** | *P. avium* | NA | Genome | KY510884 |
| **13C287_N9** | *P. serrulata* | NA | Genome | KY510885 |
| **13C287_N10** | *P. serrulata* | NA | Genome | KY510886 |
| **13C289_N11** | *P. avium* | NA | Genome | KY510887 |
| **13C289_N12** | *P. avium* | NA | Genome | KY510888 |
| **13C289_N13** | *P. avium* | NA | Genome | KY510889 |
| **13C290_N2** | *P. serrulata* | NA | Genome | KY510890 |
| **13C290_N4** | *P. serrulata* | NA | Genome | KY510891 |
| **13TF101_N33** | *P. avium* | NA | Genome | KY510892 |
| **13TF101_N34** | *P. avium* | NA | Genome | KY510893 |
| **13TF102_N34** | *P. avium* | NA | Genome | KY510894 |
| **13TF102_N36** | *P. avium* | NA | Genome | KY510895 |
| **13TF105_N28** | *P. avium* | NA | Genome | KY510896 |
| **13TF106_N6** | *P. avium* | NA | Genome | KY510897 |
| **13TF107_N7** | *P. avium* | NA | Genome | KY510898 |
| **13TF109_N35** | *P. avium* | NA | Genome | KY510899 |
| **13TF109_N36** | *P. avium* | NA | Genome | KY510900 |
| **13TF110_N23** | *P. avium* | NA | Genome | KY510901 |
| **13TF111_N28** | *P. avium* | NA | Genome | KY510902 |
| **13TF114_N5** | *P. avium* | NA | Genome | KY510903 |
| **13TF115_N23** | *P. avium* | NA | Genome | KY510904 |
| **13TF115_N29** | *P. avium* | NA | Genome | KY510905 |
| **13TF115_N30** | *P. avium* | NA | Genome | KY510906 |
| **13TF120_N7** | *P. avium* | NA | Genome | KY510907 |
| **13TF120_N8** | *P. avium* | NA | Genome | KY510908 |
| **13TF120_N9** | *P. avium* | NA | Genome | KY510909 |
| **13TF122_N26** | *P. avium* | NA | Genome | KY510910 |
| **13TF127_N29** | *P. avium* | NA | Genome | KY510911 |
| **13TF128_N7** | *P. avium* | NA | Genome | KY510912 |
| **13TF128_N8** | *P. avium* | NA | Genome | KY510913 |
| **13TF128_N9** | *P. avium* | NA | Genome | KY510914 |
| **13TF133_N5A** | *P. avium* | NA | Genome | KY510915 |
| **13TF133_N5B** | *P. avium* | NA | Genome | KY510916 |
| **13TF136_N6** | *P. cerasus* | NA | Genome | KY510917 |
| **13TF136_N7** | *P. cerasus* | NA | Genome | KY510918 |
| **13TF169_N11** | *P. salicina* | NA | Genome | KY510919 |
| **PF** | *P. domestica* | France | CP, MP | HQ267856 |
| **V590** | *P. avium* | France | CP, MP | HQ267857 |
| **HPPSC** | *P. cerasus* | India | MP | FR718887 |
| **HPSK25** | *P. avium* cv. Stella | India | MP | FR718888 |
| **JKSN28** | *P. avium* | India | MP | FR718889 |
| **JKSN29** | *P. avium* | India | MP | FR718890 |
| **WK** | *P.cerasifera* | Australia | CP, RdRp, MP | LN879388 |
| **WK** | *P. cerasifera* | Australia | MP | LN879389 |
| **JKSNM** | *P. avium* cv. Manigam | India | CP | FN669547 |
| **JKSPMi** | *P. avium* cv. Mishri | India | CP | FN669548 |
| **HPPSC** | *P. cerasus* | India | CP | FN669549 |
| **Vs2-1** | *P. avium* cv. Summit | Japan | CP | AB181355 |

a: NA, not available; b: CP, coat protein; MP, movement protein; RdRp, RNA-dependent RNA polymerase.
