# Supplementary material for: Further insight into genetic variation and haplotype diversity of Cherry virus A from China
Source: PLoS One. 2017 Oct 11;12(10):e0186273. doi: 10.1371/journal.pone.0186273 (PMC5636130; doi:10.1371/journal.pone.0186273)
Supplement: S2 Table — (DOC) [file pone.0186273.s002.doc]

**Supporting Information**

**Further Insight to Genetic Variation and Haplotype Diversity of *Cherry virus A* from China**

Rui Gao^1¶^, Yunxiao Xu^1¶^, Thierry Candresse^2^, Zhen He^3^, Shifang Li^1^, Yuxin Ma^1,2^, Meiguang Lu^1^*

^1^ State Key Laboratory for Biology of Plant Diseases and Insect Pests, Institute of Plant Protection, Chinese Academy of Agricultural Sciences, Beijing, China;

^2^ UMR 1332 BFP, INRA, Univ. Bordeaux, CS20032, 33882 Villenave d’Ornon Cedex, France;

^3^ School of Horticulture and Plant Protection, Yangzhou University, Yangzhou, Jiangsu, China.

^¶^These authors contributed equally to this work.

*Corresponding author:

Meiguang Lu ([mglu@ippcaas.cn](mailto:mglu@ippcaas.cn))

**S2 Table. Oligonucleotide primers used for amplification of the CP, RdRp, and MP CVA gene regions used in** this study

| **Primer** | **Sequence (5′- 3′)** | **Location** | **Product size (bp)** | **Reference** |
| --- | --- | --- | --- | --- |
| CVA-CP F | TGTTGGGGCACAGTTTCAAG | 5999-6018 | 1184 | X82547 |
| CVA-CP R | TGATTGGTGACGGTGAAGGA | 7182-7163 |
| CVA-RdRp F | TTGGTGTTGGATGGAGTAAGC | 3698-3718 | 1195 | X82547 |
| CVA-RdRp R | CTGGTGAGATTGTAATGGTGTTC | 4892-4870 |
| CVA-MP F | TGTCGATCATACCAGTCAAG | 5401-5420 | 707 | X82547 |
| CVA-Mp R | GTCAATGAGTGCCACAGTT | 6107-6089 |
