# Supplementary material for: Further insight into genetic variation and haplotype diversity of Cherry virus A from China
Source: PLoS One. 2017 Oct 11;12(10):e0186273. doi: 10.1371/journal.pone.0186273 (PMC5636130; doi:10.1371/journal.pone.0186273)
Supplement: S3 Table — (DOC) [file pone.0186273.s003.doc]

**Supporting Information**

**Further Insight to Genetic Variation and Haplotype Diversity of *Cherry virus A* from China**

Rui Gao^1¶^, Yunxiao Xu^1¶^, Thierry Candresse^2^, Zhen He^3^, Shifang Li^1^, Yuxin Ma^1,2^, Meiguang Lu^1^*

^1^ State Key Laboratory for Biology of Plant Diseases and Insect Pests, Institute of Plant Protection, Chinese Academy of Agricultural Sciences, Beijing, China;

^2^ UMR 1332 BFP, INRA, Univ. Bordeaux, CS20032, 33882 Villenave d’Ornon Cedex, France;

^3^ School of Horticulture and Plant Protection, Yangzhou University, Yangzhou, Jiangsu, China.

^¶^These authors contributed equally to this work.

*Corresponding author:

Meiguang Lu ([mglu@ippcaas.cn](mailto:mglu@ippcaas.cn))

**S3 Table. Oligonucleotide primers used to amplify the complete genome sequence of the ChYT52 isolate of CVA.**

| **Primer** | **Sequence (5′- 3′)** | **Location** | **Product size (bp)** | **Reference** |
| --- | --- | --- | --- | --- |
| 5′ RACE outer R | AGAAGTCACCAATCAACAGAAG | 1147-1126 | - | X82547 |
| 3′ RACE outer R | CCAACAGACTCAAGAAGACCTAT | 872-850 |
| CVA2058F | GTCTTCTTGAGTCTGTTGGA | 854-873 | 2058 | X82547 |
| CVA2058R | TATCACCTGTCACTGCTATG | 2911-2892 |
| CVA731F | TTGCTGGCTCTGGTAAATCCT | 2510-2530 | 731 | X82547 |
| CVA731R | GGTGCTTCCTTGTGCTTCATT | 3240-3220 |
| CVA2003F | GCATAGCAGTGACAGGTGATAC | 2891-2912 | 2003 | X82547 |
| CVA2003R | TCTGGTGAGATTGTAATGGTGTTC | 4893-4870 |  |
| CVA2477F | ACTTCACTCGCTCTCCAACT | 4667-4686 | 2477 | X82547 |
| CVA2477R | AATCCAAGACCTATGCCATCATC | 7143-7121 |  |
| CVA7124F | GATGGCATAGGTCTTGGATT | 7124-7143 | - | X82547 |
| Oligo (dT) | TTTTTTTTTTTTTTTTTT |  |  |  |
