# Supplementary material for: Further insight into genetic variation and haplotype diversity of Cherry virus A from China
Source: PLoS One. 2017 Oct 11;12(10):e0186273. doi: 10.1371/journal.pone.0186273 (PMC5636130; doi:10.1371/journal.pone.0186273)
Supplement: S5 Table — (DOC) [file pone.0186273.s005.doc]

**Supporting Information**

**Further Insight to Genetic Variation and Haplotype Diversity of *Cherry virus A* from China**

Rui Gao^1¶^, Yunxiao Xu^1¶^, Thierry Candresse^2^, Zhen He^3^, Shifang Li^1^, Yuxin Ma^1,2^, Meiguang Lu^1^*

^1^ State Key Laboratory for Biology of Plant Diseases and Insect Pests, Institute of Plant Protection, Chinese Academy of Agricultural Sciences, Beijing, China;

^2^ UMR 1332 BFP, INRA, Univ. Bordeaux, CS20032, 33882 Villenave d’Ornon Cedex, France;

^3^ School of Horticulture and Plant Protection, Yangzhou University, Yangzhou, Jiangsu, China.

^¶^These authors contributed equally to this work.

*Corresponding author:

Meiguang Lu ([mglu@ippcaas.cn](mailto:mglu@ippcaas.cn))

**S5 Table. Number of RdRp gene-specific haplotypes present in 31 cherry RNA samples analyzed in this study and avaliabal isolates in GenBank**

| **Haplotypes** | **Sequences number** | **Sequences/clones** | **Host** | **Selected number** |
| --- | --- | --- | --- | --- |
| Hap_1 | 1 | ChDL3-6r | *P. avium* | 1 |
| Hap_2 | 10 | ChDL3-7r; ChDL9-8r; ChYT38-6r ; ChYT37-8r; ChYT54-2r,8r; ChYT58-2r,3r,7r; ChYT59-3r | *P. avium* | 7 |
| Hap_3 | 1 | ChDL3-8r | *P. avium* | 1 |
| Hap_4 | 3 | ChDL4-4r,5r,6r | *P. avium* | 1 |
| Hap_5 | 5 | ChDL5-7r,12r; ChDL6-1r,2r,6r | *P. avium* | 2 |
| Hap_6 | 1 | ChDL5-9r | *P. avium* | 1 |
| Hap_7 | 2 | ChDL7-5r,82r | *P. avium* | 1 |
| Hap_8 | 1 | ChDL7-81r | *P. avium* | 1 |
| Hap_9 | 1 | ChDL9-1r | *P. avium* | 1 |
| Hap_10 | 1 | ChDL9-7r | *P. avium* | 1 |
| Hap_11 | 1 | ChTA10-1r | *P. avium* | 1 |
| Hap_12 | 1 | ChTA10-4r | *P. avium* | 1 |
| Hap_13 | 1 | ChTA11-1r | *P. avium* | 1 |
| Hap_14 | 1 | ChTA11-3r | *P. avium* | 1 |
| Hap_15 | 1 | ChTA11-8r | *P. avium* | 1 |
| Hap_16 | 1 | ChTA12-2r | *P. avium* | 1 |
| Hap_17 | 1 | ChTA12-6r | *P. avium* | 1 |
| Hap_18 | 1 | ChTA12-7r | *P. avium* | 1 |
| Hap_19 | 3 | ChBJ14-3r,7r,8r | *P. avium* | 1 |
| Hap_20 | 1 | ChBJ17-2r | *P. avium* | 1 |
| Hap_21 | 1 | ChBJ17-3r | *P. avium* | 1 |
| Hap_22 | 1 | ChBJ18-1r | *P. avium* | 1 |
| Hap_23 | 2 | ChBJ18-3r; ChBJ23-5r | *P. avium* | 2 |
| Hap_24 | 1 | ChBJ18-6r | *P. avium* | 1 |
| Hap_25 | 1 | ChBJ22-4r | *P. avium* | 1 |
| Hap_26 | 2 | ChBJ22-7r,8r | *P. avium* | 1 |
| Hap_27 | 1 | ChBJ23-3r | *P. avium* | 1 |
| Hap_28 | 1 | ChBJ23-7r | *P. avium* | 1 |
| Hap_29 | 1 | ChYT30-1r | *P. avium* | 1 |
| Hap_30 | 2 | ChYT30-5r,6r | *P. avium* | 1 |
| Hap_31 | 1 | ChYT31-3r | *P. avium* | 1 |
| Hap_32 | 1 | ChYT31-4r | *P. avium* | 1 |
| Hap_33 | 1 | ChYT31-5r | *P. avium* | 1 |
| Hap_34 | 1 | ChYT34-1r | *P. avium* | 1 |
| Hap_35 | 1 | ChYT34-2r | *P. avium* | 1 |
| Hap_36 | 1 | ChYT34-3r | *P. avium* | 1 |
| Hap_37 | 1 | ChYT35-6r | *P. avium* | 1 |
| Hap_38 | 1 | ChYT35-7r | *P. avium* | 1 |
| Hap_39 | 1 | ChYT35-8r | *P. avium* | 1 |
| Hap_40 | 1 | ChYT36-3r | *P. avium* | 1 |
| Hap_41 | 1 | ChYT36-5r | *P. avium* | 1 |
| Hap_42 | 1 | ChYT37-2r | *P. avium* | 1 |
| Hap_43 | 1 | ChYT37-4r | *P. avium* | 1 |
| Hap_44 | 1 | ChYT38-7r | *P. avium* | 1 |
| Hap_45 | 1 | ChYT38-3r | *P. avium* | 1 |
| Hap_46 | 2 | ChYT39-3r,5r | *P. avium* | 1 |
| Hap_47 | 1 | ChYT43-1r | *P. avium* | 1 |
| Hap_48 | 1 | ChYT43-6r | *P. avium* | 1 |
| Hap_49 | 1 | ChYT43-8r | *P. avium* | 1 |
| Hap_50 | 1 | ChYT50-2r | *P. avium* | 1 |
| Hap_51 | 1 | ChYT50-4r | *P. avium* | 1 |
| Hap_52 | 1 | ChYT50-7r | *P. avium* | 1 |
| Hap_53 | 1 | ChYT51-5r | *P. avium* | 1 |
| Hap_54 | 1 | ChYT51-6r | *P. avium* | 1 |
| Hap_55 | 1 | ChYT51-7r | *P. avium* | 1 |
| Hap_56 | 1 | ChYT52-1r | *P. avium* | 1 |
| Hap_57 | 2 | ChYT52-3r,6r | *P. avium* | 1 |
| Hap_58 | 1 | ChYT54-5r | *P. avium* | 1 |
| Hap_59 | 1 | ChYT55-1r | *P. avium* | 1 |
| Hap_60 | 1 | ChYT55-5r | *P. avium* | 1 |
| Hap_61 | 1 | ChYT55-8r | *P. avium* | 1 |
| Hap_62 | 1 | ChYT56-3r | *P. avium* | 1 |
| Hap_63 | 1 | ChYT59-5r | *P. avium* | 1 |
| Hap_64 | 1 | ChYT59-8r | *P. avium* | 1 |
| Hap_65 | 3 | ChBJ17-6r; KT285841.1; KT310083.1 | *P. avium* | 3 |
| Hap_66 | 1 | KX370827.1 | *P. avium* | 1 |
| Hap_67 | 1 | KY286055.1 | *P. mume* | 1 |
| Hap_68 | 1 | KY445749.1 | *P. mume* | 1 |
| Hap_69 | 1 | KY510845.1 | *P. cerasus* | 1 |
| Hap_70 | 1 | KY510846.1 | *P. serrulata* | 1 |
| Hap_71 | 1 | KY510847.1 | *P. serrulata* | 1 |
| Hap_72 | 1 | KY510848.1 | *P. serrulata* | 1 |
| Hap_73 | 1 | KY510849.1 | *P. avium* | 1 |
| Hap_74 | 1 | KY510850.1 | *P. avium* | 1 |
| Hap_75 | 1 | KY510851.1 | *P. serrulata* | 1 |
| Hap_76 | 6 | KY510852.1 | *P. serrulata* | 1 |
|  |  | KY510855.1 | *P. avium* | 1 |
|  |  | KY510880.1 | *P. persica* | 1 |
|  |  | KY510884.1; KY510915.1 | *P. avium* | 0 |
|  |  | KY510918.1 | *P. cerasus* | 1 |
| Hap_77 | 1 | KY510853.1 | *P. avium* | 1 |
| Hap_78 | 2 | KY510854.1 | *P. avium* | 1 |
|  |  | KY510899.1 | *P. avium* | 0 |
| Hap_79 | 2 | KY510856.1 | *P. avium* | 1 |
|  |  | KY510916.1 | *P. avium* | 0 |
| Hap_80 | 1 | KY510857.1 | *P. avium* | 1 |
| Hap_81 | 1 | KY510858.1 | *P. avium* | 1 |
| Hap_82 | 1 | KY510859.1 | *P. avium* | 1 |
| Hap_83 | 1 | KY510860.1 | *P. avium* | 1 |
| Hap_84 | 1 | KY510861.1 | *P. serrulata* | 1 |
| Hap_85 | 1 | KY510862.1 | *P. serrulata* | 1 |
| Hap_86 | 1 | KY510863.1 | *P. serrulata* | 1 |
| Hap_87 | 1 | KY510864.1 | *P. serrulata* | 1 |
| Hap_88 | 1 | KY510865.1 | *P. serrulata* | 1 |
| Hap_89 | 1 | KY510866.1 | *P. serrulata* | 1 |
| Hap_90 | 1 | KY510867.1 | *P. serrulata* | 1 |
| Hap_91 | 1 | KY510868.1 | *P. avium* | 1 |
| Hap_92 | 2 | KY510872.1 | *P. avium* | 1 |
|  |  | KY510869.1 | *P. avium* | 0 |
| Hap_93 | 1 | KY510870.1 | *P. avium* | 1 |
| Hap_94 | 2 | KY510871.1 | *P. avium* | 1 |
|  |  | KY510904.1 | *P. avium* | 0 |
| Hap_95 | 1 | KY510873.1 | *P. armeniaca* | 1 |
| Hap_96 | 1 | KY510874.1 | *P. mume* | 1 |
| Hap_97 | 4 | KY510875.1 | *P. armeniaca* | 1 |
|  |  | KY510878.1 | *P. avium* | 1 |
|  |  | KY510881.1; KY510888.1 | *P. avium* | 0 |
| Hap_98 | 1 | KY510876.1 | *P. armeniaca* | 1 |
| Hap_99 | 3 | KY510877.1 | *P. avium* | 1 |
|  |  | KY510883.1; KY510887.1 | *P. avium* | 0 |
| Hap_100 | 2 | KY510879.1 | *P. avium* | 1 |
|  |  | KY510889.1 | *P. avium* | 0 |
| Hap_101 | 1 | KY510882.1 | *P. avium* | 1 |
| Hap_102 | 1 | KY510885.1 | *P. serrulata* | 1 |
| Hap_103 | 1 | KY510886.1 | *P. serrulata* | 1 |
| Hap_104 | 1 | KY510890.1 | *P. serrulata* | 1 |
| Hap_105 | 1 | KY510891.1 | *P. serrulata* | 1 |
| Hap_106 | 1 | KY510892.1 | *P. avium* | 1 |
| Hap_107 | 2 | KY510893.1 | *P. avium* | 1 |
|  |  | KY510901.1 | *P. avium* | 0 |
| Hap_108 | 1 | KY510894.1 | *P. avium* | 1 |
| Hap_109 | 1 | KY510895.1 | *P. avium* | 1 |
| Hap_110 | 1 | KY510896.1 | *P. avium* | 1 |
| Hap_111 | 1 | KY510897.1 | *P. avium* | 1 |
| Hap_112 | 2 | KY510898.1 | *P. avium* | 1 |
|  |  | KY510900.1 | *P. avium* | 0 |
| Hap_113 | 1 | KY510902.1 | *P. avium* | 1 |
| Hap_114 | 1 | KY510903.1 | *P. avium* | 1 |
| Hap_115 | 1 | KY510905.1 | *P. avium* | 1 |
| Hap_116 | 1 | KY510906.1 | *P. avium* | 1 |
| Hap_117 | 1 | KY510907.1 | *P. avium* | 1 |
| Hap_118 | 1 | KY510908.1 | *P. avium* | 1 |
| Hap_119 | 1 | KY510909.1 | *P. avium* | 1 |
| Hap_120 | 1 | KY510910.1 | *P. avium* | 1 |
| Hap_121 | 1 | KY510911.1 | *P. avium* | 1 |
| Hap_122 | 1 | KY510912.1 | *P. avium* | 1 |
| Hap_123 | 1 | KY510913.1 | *P. avium* | 1 |
| Hap_124 | 1 | KY510914.1 | *P. avium* | 1 |
| Hap_125 | 1 | KY510917.1 | *P.cerasus* | 1 |
| Hap_126 | 1 | KY510919.1 | *P. salicina* | 1 |
| Hap_127 | 1 | X82547.1 | *P. avium* | 1 |
| Hap_128 | 1 | FN691959.1 | *P. avium* | 1 |
| Hap_129 | 1 | LN879388.1 | *P. avium* | 1 |
| Hap_130 | 1 | LC125634.1 | *P. avium* | 1 |
| Hap_131 | 1 | KU215411.1 | *P. avium* | 1 |
| Hap_132 | 1 | KU215410.1 | *Cherry* | 1 |
| Hap_133 | 1 | KU131205.1 | *P. avium* | 1 |
| **Tatal** | **175** |  |  | **147** |
