# Supplementary material for: Further insight into genetic variation and haplotype diversity of Cherry virus A from China
Source: PLoS One. 2017 Oct 11;12(10):e0186273. doi: 10.1371/journal.pone.0186273 (PMC5636130; doi:10.1371/journal.pone.0186273)
Supplement: S7 Table — (DOC) [file pone.0186273.s007.doc]

**Supporting Information**

**Further Insight to Genetic Variation and Haplotype Diversity of *Cherry virus A* from China**

Rui Gao^1¶^, Yunxiao Xu^1¶^, Thierry Candresse^2^, Zhen He^3^, Shifang Li^1^, Yuxin Ma^1,2^, Meiguang Lu^1^*

^1^ State Key Laboratory for Biology of Plant Diseases and Insect Pests, Institute of Plant Protection, Chinese Academy of Agricultural Sciences, Beijing, China;

^2^ UMR 1332 BFP, INRA, Univ. Bordeaux, CS20032, 33882 Villenave d’Ornon Cedex, France;

^3^ School of Horticulture and Plant Protection, Yangzhou University, Yangzhou, Jiangsu, China.

^¶^These authors contributed equally to this work.

*Corresponding author:

Meiguang Lu ([mglu@ippcaas.cn](mailto:mglu@ippcaas.cn))

**S7 Table. Accession numbers of the different CP, RdRp, and MP clones obtained from GenBank used** in this study.

| **Clones** | **Gene** | **AccessionNo.** | **Clones** | **Gene** | **Accession No.** |
| --- | --- | --- | --- | --- | --- |
| ChDL3-2c | CP | KY861857 | ChYT50-4r | RdRp | KY861896 |
| ChDL5-21c | CP | KY861858 | ChYT51-6r | RdRp | KY861897 |
| ChTA10-5c | CP | KY861859 | ChY-52-6r | RdRp | KY861898 |
| ChTA11-4c | CP | KY861860 | ChYT55-1r | RdRp | KY861899 |
| ChTA12-2c | CP | KY861861 | ChYT55-8r | RdRp | KY861900 |
| ChTA12-5c | CP | KY861862 | ChDL3-8m | MP | KY861901 |
| ChBJ17-1c | CP | KY861863 | ChDL4-5m | MP | KY861902 |
| ChBJ23-16c | CP | KY861864 | ChDL4-7m | MP | KY861903 |
| ChYT30-6c | CP | KY861865 | ChDL5-2m | MP | KY861904 |
| ChYT31-3c | CP | KY861866 | ChDL7-5m | MP | KY861905 |
| ChYT38-2c | CP | KY861867 | ChDL9-1m | MP | KY861906 |
| ChYT39-3c | CP | KY861868 | ChTA10-3m | MP | KY861907 |
| ChYT50-1c | CP | KY861869 | ChTA10-7m | MP | KY861908 |
| ChYT50-8c | CP | KY861870 | ChTA11-8m | MP | KY861909 |
| ChYT51-2c | CP | KY861871 | ChTA12-1m | MP | KY861910 |
| ChYT52-1c | CP | KY861872 | ChBJ14-1m | MP | KY861911 |
| ChYT56-2c | CP | KY861873 | ChBJ17-8m | MP | KY861912 |
| ChYT58-2c | CP | KY861874 | ChYT30-3m | MP | KY861913 |
| ChDL3-6r | RdRp | KY861875 | ChYT31-7m | MP | KY861914 |
| ChDL5-9r | RdRp | KY861876 | ChYT34-2m | MP | KY861915 |
| ChTA10-1r | RdRp | KY861877 | ChYT34-5m | MP | KY861916 |
| ChTA11-1r | RdRp | KY861878 | ChYT35-3m | MP | KY861917 |
| ChTA11-3r | RdRp | KY861879 | ChYT35- 8m | MP | KY861918 |
| ChTA12-6r | RdRp | KY861880 | ChYT36-2m | MP | KY861919 |
| ChBJ14-3r | RdRp | KY861881 | ChYT36-5m | MP | KY861920 |
| ChBJ17-2r | RdRp | KY861882 | ChYT52-3m, | MP | KY861921 |
| ChYT30-1r | RdRp | KY861883 | ChYT55-1m | MP | KY861922 |
| ChYT31-4r | RdRp | KY861884 | ChYT55-7m | MP | KY861923 |
| ChYT34-1r | RdRp | KY861885 | ChYT56-1m | MP | KY861924 |
| ChYT34-3r | RdRp | KY861886 | ChYT58-5m | MP | KY861925 |
| ChYT35-7r | RdRp | KY861887 | ChYT50-5m | MP | MF991133 |
| ChYT36-3r | RdRp | KY861888 | ChYT50-6m | MP | MF991134 |
| ChYT36-5r | RdRp | KY861889 | ChYT39-7m | MP | MF991132 |
| ChYT37-2r | RdRp | KY861890 | ChYT30-5m | MP | MF991131 |
| ChYT38-7r | RdRp | KY861891 | ChDL6-6m | MP | MF991126 |
| ChYT39-5r | RdRp | KY861892 | ChDL7-6m | MP | MF991127 |
| ChYT43-1r | RdRp | KY861893 | ChDL9-3m | MP | MF991128 |
| ChYT43-8r | RdRp | KY861894 | ChTA12-8m | MP | MF991129 |
| ChYT50-2r | RdRp | KY861895 | ChBJ14-2m | MP | MF991130 |
