# Supplementary material for: Further insight into genetic variation and haplotype diversity of Cherry virus A from China
Source: PLoS One. 2017 Oct 11;12(10):e0186273. doi: 10.1371/journal.pone.0186273 (PMC5636130; doi:10.1371/journal.pone.0186273)
Supplement: S8 Table — a: P-values determined for eachof the seven different programs (RDP, GENECONV, BootScan, MaxChi, Chimaera, SiSCan, and 3Seq) implemented in RDP 4.0 software. (DOC) [file pone.0186273.s008.doc]

**Supporting Information**

**Further Insight to Genetic Variation and Haplotype Diversity of *Cherry virus A* from China**

Rui Gao^1¶^, Yunxiao Xu^1¶^, Thierry Candresse^2^, Zhen He^3^, Shifang Li^1^, Yuxin Ma^1,2^, Meiguang Lu^1^*

^1^ State Key Laboratory for Biology of Plant Diseases and Insect Pests, Institute of Plant Protection, Chinese Academy of Agricultural Sciences, Beijing, China;

^2^ UMR 1332 BFP, INRA, Univ. Bordeaux, CS20032, 33882 Villenave d’Ornon Cedex, France;

^3^ School of Horticulture and Plant Protection, Yangzhou University, Yangzhou, Jiangsu, China.

^¶^These authors contributed equally to this work.

*Corresponding author:

Meiguang Lu ([mglu@ippcaas.cn](mailto:mglu@ippcaas.cn))

**S8 Table. Recombination events detected in the RdRp and MP gene datasets using RDP 4.0**

| **Region** | **RdRp** | | **MP** | | | |
| --- | --- | --- | --- | --- | --- | --- |
| **Recombinant** | ChBJ17-2r_*P. avium* | ChYT38-7r_*P. avium* | ChDL6-6m_*P. avium* | ChTA11-8m_*P. avium* | ChYT34-2m_*P. avium* | ChYT50-5m_*P. avium* |
| Major Parent | 13TF120_N7_*P. avium* | 13TF128_N7_*P. avium* | 13TF136_N7_*P. cerasus* | 13TF136_N7_*P. cerasus* | 13TF136_N7_*P. cerasus* | ChYT39-7m_*P. avium* |
| Minor Parent | 13C222_N8_*P. avium* | 13TF128_N9_*P. avium* | ChYT39-7m_*P. avium* | ChYT39-7m_*P. avium* | ChYT39-7m_*P. avium* | 13TF136_N7_*P. cerasus* |
| **Beginning breakpoint (nt)** | 799 | 796 | 17 | 425 | 425 | 282 |
| **Ending breakpoint (nt)** | 380 | 270 | 541 | 495 | 495 | 495 |
| ***p-Value*^a^** | | | | | | |
| RDP | 1.279×10^-14^ | 9.470×10^-12^ | 3.056×10^-1^ | 3.056×10^-1^ | 3.056×10^-1^ | 3.056×10^-1^ |
| GENECONV | 9.990×10^-13^ | 4.991×10^-9^ | 1.946×10^-2^ | 1.946×10^-2^ | 1.946×10^-2^ | 1.946×10^-2^ |
| BootScan | 1.872×10^-15^ | 8.808×10^-11^ | n.d. | n.d. | n.d. | 2.333×10^-4^ |
| MaxChi | 3.776×10^-13^ | 4.074×10^-10^ | 3.161×10^-6^ | 3.161×10^-6^ | 3.161×10^-6^ | 3.161×10^-6^ |
| Chimaera | 4.061×10^-13^ | 3.862×10^-10^ | 1.711×10^-6^ | 1.711×10^-6^ | 1.711×10^-6^ | 1.711×10^-6^ |
| SiSCan | 6.817×10^-18^ | 5.749×10^-8^ | 9.667×10^-6^ | 9.667×10^-6^ | 9.667×10^-6^ | 9.667×10^-6^ |
| 3Seq | 1.530×10^-28^ | 1.700×10^-17^ | 5.577×10^-7^ | 5.577×10^-7^ | 5.577×10^-7^ | 5.577×10^-7^ |

^a^: *P-values* determined for eachof the seven different programs (RDP, GENECONV, BootScan, MaxChi, Chimaera, SiSCan, and 3Seq) implemented in

RDP 4.0 software.
