# Supplementary material for: Further insight into genetic variation and haplotype diversity of Cherry virus A from China
Source: PLoS One. 2017 Oct 11;12(10):e0186273. doi: 10.1371/journal.pone.0186273 (PMC5636130; doi:10.1371/journal.pone.0186273)
Supplement: S9 Table — a: P-values determined for eachof the seven different programs (RDP, GENECONV, BootScan, MaxChi, Chimaera, SiSCan, and 3Seq) implemented in RDP 4.0 software. *: These isolates may be actual recombinants. (DOC) [file pone.0186273.s009.doc]

**Supporting Information**

**Further Insight to Genetic Variation and Haplotype Diversity of *Cherry virus A* from China**

Rui Gao^1¶^, Yunxiao Xu^1¶^, Thierry Candresse^2^, Zhen He^3^, Shifang Li^1^, Yuxin Ma^1,2^, Meiguang Lu^1^*

^1^ State Key Laboratory for Biology of Plant Diseases and Insect Pests, Institute of Plant Protection, Chinese Academy of Agricultural Sciences, Beijing, China;

^2^ UMR 1332 BFP, INRA, Univ. Bordeaux, CS20032, 33882 Villenave d’Ornon Cedex, France;

^3^ School of Horticulture and Plant Protection, Yangzhou University, Yangzhou, Jiangsu, China.

^¶^These authors contributed equally to this work.

*Corresponding author:

Meiguang Lu ([mglu@ippcaas.cn](mailto:mglu@ippcaas.cn))

**S9 Table. Potential recombination events detected in the CP and MP gene datasets using RDP 4.0**

| **Region** | **CP** | | | | | | **MP** | | | | |
| --- | --- | --- | --- | --- | --- | --- | --- | --- | --- | --- | --- |
| **Recombinant** | 13C222_N8_*P. avium* | 13C224_N6_*P. serrulata* | 13C231_N4_ *P. serrulata* | 13TF105_N28_ *P. avium* | 13TF136_N7_*P. cerasus* | ChTA12-2c_ *P. avium* | ChDL4-7m_ *P. avium* | | ChYT39-6m_*P. avium* | | ChYT50-6m_*P. avium* |
| Major Parent | 13C206_N12_*P. serrulata* | 13C206_N12_*P. serrulata* | ChYT59-1c_*P. avium* | ChYT59-1c_*P. avium* | ChYT59-1c_*P. avium* | ChYT59-1c_*P. avium* | *ChYT36-5m_*P. avium* | | *ChYT36-5m_*P. avium* | | 13TF109_N36_*P. avium* |
| Minor Parent | *Vs2-1 | *Vs2-1 | *Vs2-1 | *Vs2-1 | *Vs2-1 | *Vs2-1 | *ChDL4-5m_*P. avium* | | *ChYT39-7m_*P. avium* | | 13TF105_N28_ *P. avium* |
| **Beginning breakpoint (nt)** | 302 | 194 | 21 | 530 | 530 | 594 | 562 | | 568 | | 128 |
| **Ending breakpoint (nt)** | 602 | 613 | 388 | 349 | 349 | 240 | 276 | | 220 | | 292 |
| ***p-Value*^a^** | | | | | | | | | | | |
| RDP | 1.379×10^-3^ | 1.379×10^-3^ | 3.461×10^-1^ | 3.462×10^-1^ | 3.462×10^-1^ | 3.462×10^-1^ | 1.043×10^-5^ | 6.060×10^-4^ | | 5.136×10^-5^ | |
| GENECONV | 3.727×10^-4^ | 3.727×10^-4^ | 1.573×10^-3^ | 1.573×10^-3^ | 1.573×10^-3^ | 1.573×10^-3^ | 2.005×10^-3^ | 1.447×10^-2^ | | 1.208×10^-3^ | |
| BootScan | 1.558×10^-7^ | 1.558×10^-7^ | 4.438×10^-5^ | 4.438×10^-5^ | 4.438×10^-5^ | 4.438×10^-5^ | 6.154×10^-5^ | 3.734×10^-3^ | | 5.229×10^-5^ | |
| MaxChi | 5.475×10^-7^ | 5.475×10^-7^ | 5.284×10^-4^ | 5.284×10^-4^ | 5.284×10^-4^ | 5.284×10^-4^ | 8.135×10^-7^ | 2.395×10^-2^ | | 2.980×10^-3^ | |
| Chimaera | 1.563×10^-4^ | 1.563×10^-4^ | 3.166×10^-5^ | 3.166×10^-5^ | 3.166×10^-5^ | 3.166×10^-5^ | 4.131×10^-4^ | 2.070×10^-2^ | | 2.667×10^-3^ | |
| SiSCan | 1.048×10^-12^ | 1.048×10^-12^ | 4.223×10^-7^ | 4.223×10^-7^ | 4.223×10^-7^ | 4.223×10^-7^ | 1.509×10^-2^ | 2.961×10^-3^ | | 1.255×10^-5^ | |
| 3Seq | 1.950×10^-4^ | 1.950×10^-4^ | 2.763×10^-11^ | 2.763×10^-11^ | 2.763×10^-11^ | 2.763×10^-11^ | 1.326×10^-8^ | 5.659×10^-5^ | | 6.345×10^-7^ | |

^a^: *P-values* determined for eachof the seven different programs (RDP, GENECONV, BootScan, MaxChi, Chimaera, SiSCan, and 3Seq) implemented in RDP 4.0 software.

*: These isolates may be actual recombinants.
